# Circulating miR-let7a levels predict future diagnosis of chronic thromboembolic pulmonary hypertension

**DOI:** 10.1038/s41598-024-55223-1

**Published:** 2024-02-24

**Authors:** Franziska Kenneweg, Lukas Hobohm, Claudia Bang, Shashi K. Gupta, Ke Xiao, Sabrina Thum, Vincent Ten Cate, Steffen Rapp, Gerd Hasenfuß, Philipp Wild, Stavros Konstantinides, Rolf Wachter, Mareike Lankeit, Thomas Thum

**Affiliations:** 1 Institute of Molecular and Translational Therapeutic Strategies (IMTTS), Hannover Medical School, Hannover, Germany; 2 REBIRTH Excellence Cluster, Hannover Medical School, Hannover, Germany; 3 Department of Cardiology, University Medical Center Mainz, Mainz, Germany; 4 Center for Thrombosis and Hemostasis (CTH), University Medical Center Mainz, Mainz, Germany; 5 Preventive Cardiology and Preventive Medicine, Department of Cardiology, University Medical Center Mainz, Mainz, Germany; 6 Clinical Epidemiology and Systems Medicine, Center for Thrombosis and Hemostasis (CTH), Mainz, Germany; 7 German Cardiovascular Research Centre (DZHK), Partner Site Rhine Main, Mainz, Germany; 8 Institute of Molecular Biology (IMB), Mainz, Germany; 9 Clinic of Cardiology and Pneumology, Heart Center, University Medical Center, Goettingen, Germany; 10 Clinic and Policlinic for Cardiology, University Hospital Leipzig, Leipzig, Germany; 11 Department of Internal Medicine and Cardiology, Campus Virchow Klinikum (CVK), Charité-University Medicine Berlin, Berlin, Germany

**Keywords:** Biomarkers, Thromboembolism

## Abstract

Distinct patterns of circulating microRNAs (miRNAs) were found to be involved in misguided thrombus resolution. Thus, we aimed to investigate dysregulated miRNA signatures during the acute phase of pulmonary embolism (PE) and test their diagnostic and predictive value for future diagnosis of chronic thromboembolic pulmonary hypertension (CTEPH). Microarray screening and subsequent validation in a large patient cohort (n = 177) identified three dysregulated miRNAs as potential biomarkers: circulating miR-29a and miR-720 were significantly upregulated and miR-let7a was significantly downregulated in plasma of patients with PE. In a second validation study equal expression patterns for miR-29a and miR-let7a regarding an acute event of recurrent venous thromboembolism (VTE) or deaths were found. MiR-let7a concentrations significantly correlated with echocardiographic and laboratory parameters indicating right ventricular (RV) dysfunction. Additionally, circulating miR-let7a levels were associated with diagnosis of CTEPH during follow-up. Regarding CTEPH diagnosis, ROC analysis illustrated an AUC of 0.767 (95% CI 0.54–0.99) for miR-let7a. Using logistic regression analysis, a calculated patient-cohort optimized miR-let7a cut-off value derived from ROC analysis of ≥ 11.92 was associated with a 12.8-fold increased risk for CTEPH. Therefore, miR-let7a might serve as a novel biomarker to identify patients with haemodynamic impairment and as a novel predictor for patients at risk for CTEPH.

## Introduction

Pulmonary embolism (PE) is the third most common cardiovascular disease with an annual incidence of 39–115 per 100,000 person-years and is associated with serious short- and long-term complications including recurrence, chronic thromboembolic pulmonary hypertension (CTEPH) and death^1,2^. CTEPH is a rare disease, but is associated with high mortality rates and poor prognosis if left untreated^3,4^. In recent years, microRNAs (miRNAs) emerged as novel biomarkers and distinct patterns were found to exhibit diagnostic and prognostic value for different disease settings, including various cardiovascular diseases^5,6^. Studies have proven the existence of dysbalanced miRNA concentrations with disease-specific changes in different cell types and can thus reflect many different pathological conditions. Moreover, miRNAs are stable in the circulation (including blood, urine, saliva breast milk) and thus are promising diagnostic and prognostic markers^7,8^.

Little is known about the miRNA profiles in patients with acute PE and CTEPH. Therefore, we evaluated whether there are common miRNA expression patterns among patients suffering from acute PE as compared to healthy control patients and assessed the diagnostic and prognostic value of specific miRNAs on different pre-defined short-term and long-term outcomes.

## Results

### Screening for circulating miRNAs in plasma of PE patients and baseline characteristics

**Figure 1:**
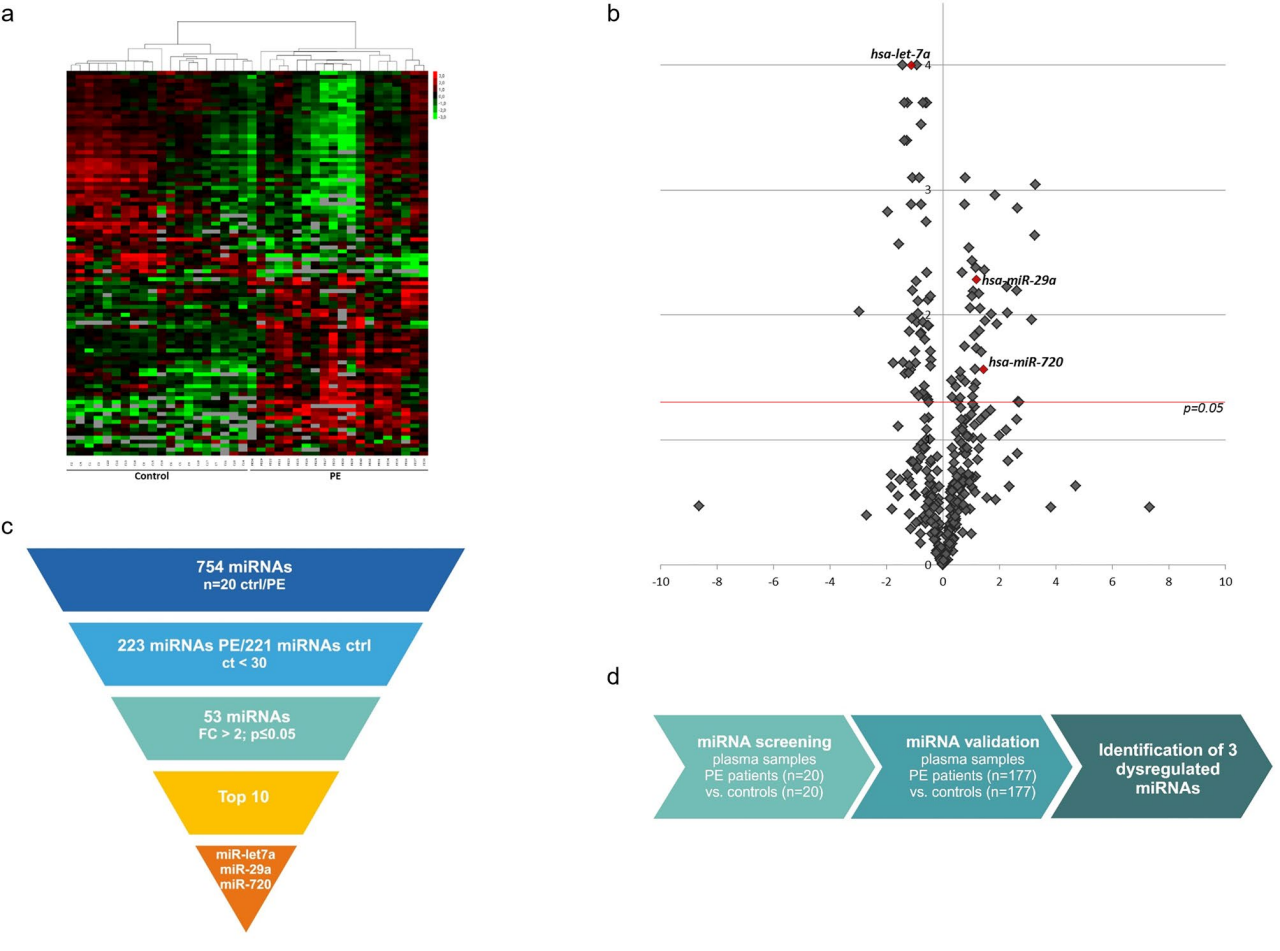
Microarray-based global expression profiling of miRNAs in patients with acute pulmonary embolism. (**a**) Heatmap of TaqMan miRNA Array and (**b**) volcano plot of deregulated miRNAs in plasma of patients with pulmonary embolism (n = 20) and sex- and age-matched healthy controls (n = 20). (**c**) Flow-chart of miRNA-screening and the study (**d**).Graphic was created with biorender. PE = pulmonary embolism.

Between October 2005 and September 2012, we included 177 patients (52.9% women; median age 70 [IQR, 52–76] years) with acute PE. The baseline characteristics of the study patients are shown in Table 1. We randomly selected 20 PE patients and 20 sex- and age-matched healthy controls and screened for significantly dysregulated circulating miRNAs in plasma of acute PE vs. healthy controls using microarray-PCR-based global expression profiling **(**Fig. 1a,b).

**Table 1:** Baseline characteristics, medical history, and initial presentation of all study patients with acute pulmonary embolism.

	All study patients (n = 177)
Sex (female)	92 (51.9%)
Age (years)	70 (52–76)
BMI (kg/m^2^)	27.7 (24.5–31.1)
**Risk factors for VTE and comorbidities**	
Unprovoked PE	102 (57.1%)
Previous PE	24 (13.4%)
Recent surgery*	29 (16.2%)
Pregnancy/post partum**	3 (1.7%)
Thrombophilia	6 (3.4%)
Hyperlipoproteinaemia	34 (19.2%)
**Symptoms and clinical findings on admission**	
Syncope	21 (11.7%)
Heart rate (bpm)	88 (75–105)
Dyspnoea	154 (86.2%)
Systolic blood pressure (mmHg)	130 (120–150)
RV dysfunction^††^ on TTE n = 108	54 (50.0%)
RV/LV ratio on CT n = 141	96 (68.0%)
Elevated troponin (hsTnT ≥ 13.9 pg/ml) n = 72	42 (58.1%)
ESC 2019 low risk	26 (14.7%)
ESC 2019 intermediate-low risk	89 (50.3%)
ESC 2019 intermediate-high risk	62 (35.0%)
GFR (ml/min/1.73 m^2^)	75.3 (58.1–93.3)
CRP (mg/l)	33 (11.1–68.1)
**Complications, treatment and short-term outcomes**	
Thrombolysis	13 (7.3%)
Adverse outcome^§§^	11 (6.2%)
PE-related death	4 (2.3%)
All-cause death	4 (2.3%)
In-hospital stay (days)	9 (6–13)
**Follow-up and Long-term Outcome**	
Longterm follow-up (days)	2968 (1186–2691)
Longterm mortality	33 (18.5%)
Recurrence of VTE	1 (0.6%)
CTEPH	6 (3.4%)

*BMI* body mass index, *DVT* deep vein thrombosis, *PE* pulmonary embolism, *ESC* Euopean Sociey of Cardiology, *Spesi* simplified Pulmonary Embolism Severity Index, *CT* computed tomography, *TTE* transthoracic echocardiography, *RVD* right ventricular dysfunction, *hsTnT* high sensitive troponin T, *NT-proBNP* N-terminal propeptide of B-type natriuretic peptide, *GFR* glomerular filtration rate, *CRP* C-reactive protein, *VTE* venous thromboembolism, *CTEPH* chronic thromboembolic pulmonary hypertension.

*Within the last 4 weeks.

**Post partum within the last 6 weeks.

^††^RV dysfunction was defined as dilatation of the right ventricle (end-diastolic diameter > 30 mm from the parasternal view, or a right ventricle/left ventricle diameter ratio > 1.0 from the subcostal or apical views), combined with right atrial hypertension (absence of the inspiratory collapse of the inferior vena cava).

^§^Low risk was defined as absence of RV dysfunction and absence of troponin elevation; intermediate risk was defined as RV dysfunction and/or troponin elevation; high risk was defined as haemodynamic instability.

^§§^Defined as mechanical ventilation, catecholamine administration, CPR or all-cause death within 30 days.

**Figure 2:**
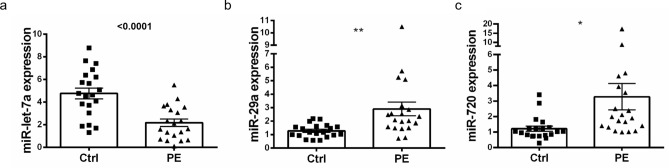
Dysregulated miRNAs in patients with acute pulmonary embolism. After stringent selection strategy 3 miRNAs were identified to be significantly deregulated in plasma of patients with pulmonary embolism (n = 20) and sex- and age-matched healthy controls (n = 20): miR-let-7a (**a**), miR-29a (**b**) and miR-720 (**c**). Ctrl healthy control, PE pulmonary embolism. data is shown as normalized values ± SEM; *p ≤ 0.05; **p ≤ 0.01; Student’s t-test of patients with PE and specific sex- and age-matched healthy controls.

Out of 754 miRNAs on the TaqMan miRNA Array 223 miRNAs were detectable in PE patients and 221 in controls with sufficient detection levels (ct < 30). After filtering steps according to stringent selection criteria (fold-change more than 2; significance level p ≤ 0.05) 53 miRNAs were significantly deregulated and were considered for further validation. We chose the top 10 dysregulated miRNAs (Supplemental Table 1) and from these 3 miRNAs showed sufficient amplification plots in plasma: miR-29a, miR-720 and miR-let7a (Fig. 1c). Of those, circulating miR-29a and miR-720 were significantly upregulated and miR-let7a was significantly downregulated (Fig. 2). Thereby, the nomenclature of miR-720 is still under debate and it seems that miR-720 is not a classical miRNA, but likely a fragment of a t-RNA.

### Presence of miR-29a, miR-720 and miR-let7a in patients with acute PE

**Figure 3:**
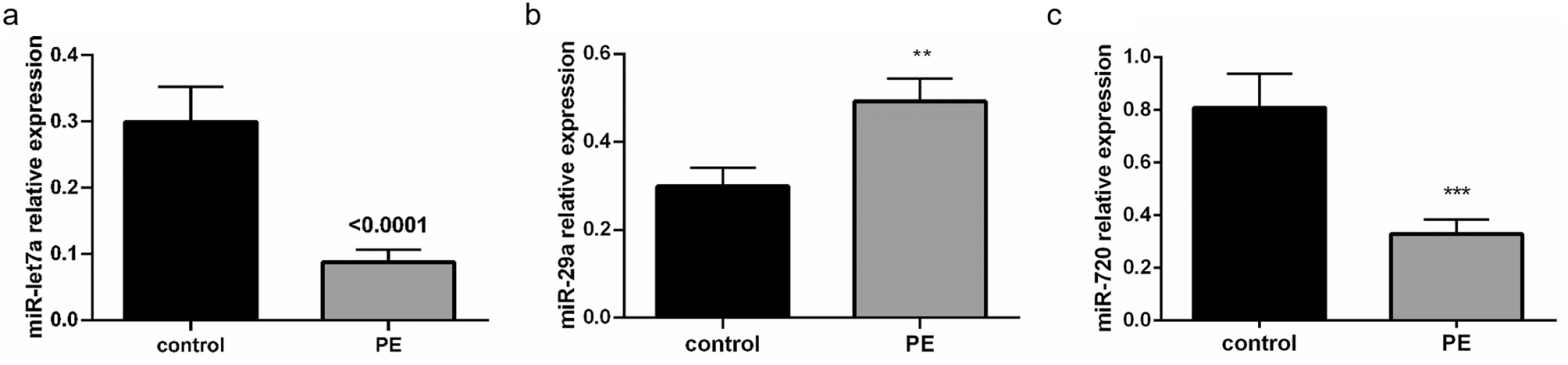
miR-let7a, miR-29a and miR-720 are dysregulated in patients with acute pulmonary embolism. The three promising miRNAs were validated in a bigger patient cohort (n = 177) by qPCR. (**a**) miR-let7a was found to be significantly decreased, (**b**) miR-29a to be significantly increased and (**c**) miR-720 to be significantly decreased in patients with pulmonary embolism (PE).; Data is shown as normalized expression to cel-miR-39 ± SEM; **p ≤ 0.01; ***p ≤ 0.001; Student´s t-test of patients with PE and specific sex- and age-matched healthy controls.

Next, we profiled these three promising miRNAs in 177 patients with confirmed acute PE and 177 sex- and age-matched healthy controls (Fig. 1d). MiR-29a levels could be detected in 158 PE patients with abundancy over a certain threshold (ct < 30) and ranged from 0.1 to 378.1 (median, 0.3 [IQR, 0.1–1.3]). MiR-720 levels were detectable in 174 PE patients and ranged from 0.1 to 6.9 (median, 0.1 [IQR, 0.1–0.2]) and miR -let7a levels in 153 PE patients ranging from − 6.1 to 19.8 (median, 10.3 [IQR, 8.8–12.3]). We confirmed that circulating miR-29a levels were significantly increased and miR-let7a levels were significantly decreased in PE patients compared to matched healthy control patients (Fig. 3a,b and supplemental Fig. 1a,b), suggesting that circulating miR-29a and miR-let7a might serve as novel biomarkers for acute PE. Circulating miR-720 levels were downregulated in PE patients (Fig. 3c) (miR-29a in 109 matched healthy controls: median, 0.11 [IQR 0.05–0.25], difference − 0.26 ± 0.69, p = 0.001; miR-720 in 167 matched healthy controls: median, 0.05 [IQR 0.02–0.23], difference 0.47 ± 1.52, p < 0.001; miR-let7a in 126 matched healthy controls: median, 0.30 [IQR, 0.00–0.25], difference 0.18 ± 0.56, p = 0.002). For miR-29a and miR-720 only one parameter in each case was found to be associated with higher levels (hyperlipoproteinaemia for miR-29a: OR 2.8 [1.2–6.5]; p = 0.013 and the history of previous PE for miR-720: OR 2.6 [1.0–6.8]; p = 0.050). Interestingly, predictors for high levels of miR-let7a were only parameters regarding right ventricular (RV) compromise such as RV/LV ratio ≥ 1 on transthoracic echocardiography (TTE) (OR 3.9 [1.4–10.8]; p = 0.009) or CT (OR 2.3 [1.0–4.9]; p = 0.042) as well as elevated troponin (hsTnT) ≥ 14 pg/ml (OR 3.6 [1.8–7.1]; p < 0.001) indicating a potential pathomechanistic role for miR-let7a in PE.

### Association between miR-29a and miR-let7a levels and adverse outcome in independent validation cohort

Next, we validated our findings in an independent patient cohort. In this cohort, 117 study participants experienced a recurrent event or died (first event recurrent VTE, n = 53; death n = 64). The median time to event was 2.6 years (interquartile range [IQR]: 0.7–3.2), and the maximum follow-up time was 7.9 years. Expression levels of miRNA-29a(-3p) were positively related to incidence of the combined endpoint (Table 2), with a 29% risk increase per standard deviation increase of expression (age and sex-adjusted HR: 1.29 [95% CI 1.06–1.55], p = 0.009). Conversely, levels of miR-let7a were inversely related to the same endpoint (age and sex-adjusted HR per standard deviation increase: 0.72 [95% CI 0.60–0.86], p = 0.0003).

**Table 2:** Prediction of an acute event of recurrent venous thromboembolism by miR-29a and miR-let7a in an independent validation cohort of individuals with acute index event.

	Hazard ratio [95% CI]	p-value
miR-29a-3p (SD)	1.29 [1.06–1.55]	0.0093
miR-let7a (SD)	0.72 [0.60–0.86]	0.0003

Results of two separate Cox regression models predicting the combined endpoint of recurrent VTE or death using standardized miRNA expression levels, adjusted for age and sex. The validation cohort subsample with miRNA sequencing comprised 181 individuals, 117 of whom experienced recurrent events or died during the follow-up period (median [IQR]: 2.6 [0.7–3.2] years, max.: 7.9 years).

*CI* confidence interval, *IQR* interquartile range, *SD* standard deviation.

### Correlation of miR-let7a levels and haemodynamic impairment in the acute phase and with diagnosis of CTEPH during follow-up

No correlation with echocardiographic and laboratory parameters indicating haemodynamic RV impairment were observed for circulating miR-29a and miR-720, but for miR-let7a (estimated systolic PA pressure on TTE, r = 0.336, p = 0.012; NT-proBNP, r = 0.216, p = 0.008), highlighting this specific miRNA to be of clinical interest. For investigation of the prognostic value of miRNAs regarding short-term outcomes, ROC analyses illustrated an AUC of 0.43 (95% CI 0.24–0.63) for miR-29a, an AUC of 0.44 (95% CI 0.24–0.64) for miR-720 and an ROC of 0.42 (95% CI 0.28–0.67) for miR-let7a with regard to an adverse outcome. Using logistic regression analyses, no miRNA or other parameter such as clinical findings (mild hypotension [OR 5.4 [0.5–57.1]; p = 0.158] or syncope [OR 1.7 [0.3–8.6]; p = 0.508]), laboratory (NT-proBNP ≥ 600 pg/ml [OR 1.2 [0.4–4.2]; p = 0.731]) or imaging parameter (RV dysfunction on CT [OR 4.0 [0.5–32.9]; p = 0.198]) was associated with short-term prognosis (adverse outcome) in normotensive patients with acute PE.

**Figure 4:**
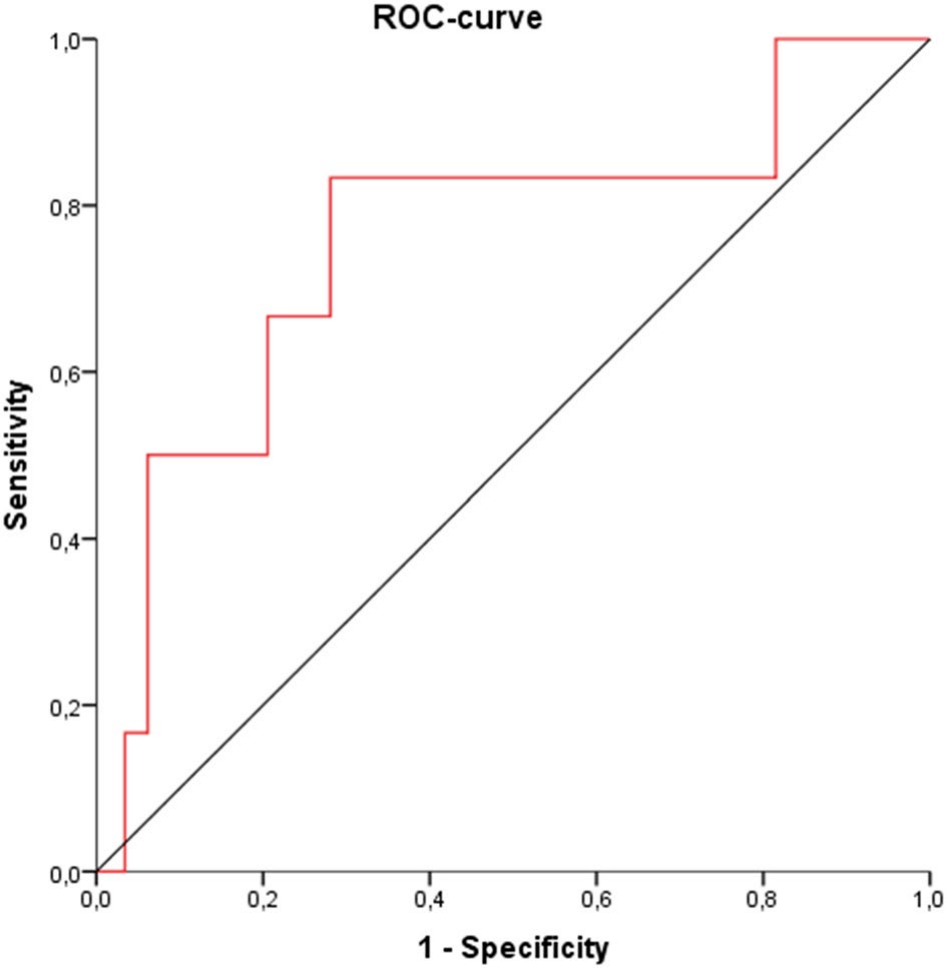
Prognostic performance (receiver operating characteristic [ROC]) of miR-let7a regarding the diagnosis of CTEPH in patients with pulmonary embolism who underwent clinical follow-up.

However, regarding the diagnosis of CTEPH (n = 6; 3.9%) during follow-up, ROC analysis illustrated a relevant AUC for miR-let7a (0.767 [95% CI 0.54–0.99]; p = 0.027]) (Fig. 4). The calculated optimal cut-off value for miR-let7a on admission of ≥ 11.92 was associated with a sensitivity of 78%, a specificity of 31%, a PPV of 7% and a NPV of 96%. By using univariate logistic regression analysis, the calculated patient-cohort optimized miR-let7a cut-off value derived from ROC analysis was associated with a 12.8-fold increased risk (1.5–112.9, p = 0.002) for a diagnosis of CTEPH during follow-up. Those results were not affected by performing a multivariable logisitic regression analysis with adjusting age and sex (OR 14.5 [1.6–131.9]; p = 0.017). Of note, miR-29a and miR-720 levels and calculated patient-cohort optimized cut-off values were not associated with an increased risk for a diagnosis of CTEPH during follow-up. The baseline characteristics of the PE patients with miR-let7a measurement are demonstrated in Table 3.

**Table 3:** Baseline characteristics, medical history, and initial presentation of 153 normotensive patients with acute pulmonary embolism stratified by miR-let7a above and below median.

	All study patients (n = 153)	MiR let7a above median (n = 78)	MiR let7a below median (n = 75)	p-value
Sex (female)	78 (51.0%)	45 (58.4%)	33 (43.4%)	0.076
Age (years)	70 (52–76)	72 (51–78)	66 (51–73)	0.068
BMI (kg/m^2^)	27.7 (24.5–31.1)	28 (25–30)	28 (24–33)	0.514
**Risk factors for VTE and comorbidities**				
Unprovoked PE	92 (60.1%)	45 (58.4%)	47 (61.8%)	0.742
Previous PE	23 (15.0%)	10 (13.0%)	13 (17.1%)	0.506
Recent surgery*	21 (13.7%)	13 (16.9%)	8 (10.5%)	0.348
Pregnancy/post partum**	3 (2.0%)	1 (1.3%)	2 (2.6%)	0.620
Thrombophilia	5 (3.3%)	1 (1.3%)	4 (5.3%)	0.209
Hyperlipoproteinaemia	34 (22.2%)	14 (18.2%)	20 (26.3%)	0.243
Chronic heart failure	21 (13.7%)	9 (11.7%)	12 (15.8%)	0.490
Coronary artery disease	22 (14.4%)	11 (14.3%)	11 (14.5%)	1.000
Chronic pulmonary disease	18 (11.8%)	6 (7.8%)	12 (15.8%)	0.140
Arterial hypertension	92 (60.1%)	44 (57.2%)	48 (63.2%)	0.510
Previous ischemic stroke	11 (7.2%)	5 (6.5%)	6 (7.9%)	0.765
Diabetes mellitus	24 (15.7%)	9 (11.7%)	15 (19.7%)	0.189
**Symptoms and clinical findings on admission**				
Syncope	18 (11.8%)	7 (9.1%)	11 (14.5%)	0.327
Heart rate (bpm)	88 (75–105)	90 (76–105)	83 (75–103)	0.474
Heart rate ≥ 100 bpm	48 (31.4%)	24 (31.2%)	24 (31.6%)	1.000
Chest pain	91 (59.5%)	41 (53.2%)	50 (65.8%)	0.139
Dyspnoea	135 (88.2%)	68 (88.3%)	67 (88.2%)	1.000
Sepsis	43 (28.1%)	20 (26.0%)	23 (30.3%)	0.593
Systolic blood pressure (mmHg)	130 (120–150)	130 (120–150)	130 (120–150)	0.484
Mild hypotension (systolic BP < 100 mmHg)	4 (2.6%)	2 (2.6%)	2 (2.6%)	1.000
Hypoxia^†^ n = 129	33 (25.6%)	14 (20.6%)	19 (31.1%)	0.225
Echocardiography performed within 48 h	91 (59.5%)	45 (58.4%)	46 (60.5%)	0.870
RV dysfunction on TTE^††^ n = 91	45 (49.5%)	27 (60.0%)	18 (39.1%)	0.060
Estimated systolic PAP n = 72	39 (32–50)	43 (35–52)	36 (25–50)	0.089
Computed tomography performed	148 (96.7%)	75 (97.4%)	73 (96.1%)	0.681
RV/LV ratio on CT n = 127	88 (69.3%)	49 (77.8%)	39 (60.9%)	0.054
Elevated troponin (hsTnT ≥ 13.9 pg/ml) n = 151	88 (58.3%)	55 (73.3%)	33 (43.4%)	** < 0.001**
Elevated NTproBNP (≥ 600 pg/ml) n = 151	75 (49.7%)	43 (57.3%)	32 (42.1%)	0.074
GFR (ml/min/1.73 m^2^)	75.3 (58.1–93.3)	73.5 (55.9–95.6)	76.4 (60.1–93.3)	0.456
CRP (mg/l)	33 (11.1–68.1)	34 (12.1–70.4)	25 (9.4–57.6)	0.350
**Scores**				
sPESI ≥ 1	80 (52.3%)	40 (51.9%)	40 (52.6%)	1.000
ESC2019^§^ (interm.-high vs. interm.-low and low-risk)	53 (34.6%)	30 (39.0%)	23 (30.3%)	0.309
**Complications, treatment and short-term outcomes**				
Administration of catecholamines	8 (5.2%)	3 (3.9%)	5 (6.6%)	0.495
Mechanical ventilation	8 (5.2%)	3 (3.9%)	5 (6.6%)	0.495
Cardio-pulmonary resuscitation	2 (1.3%)	0 (0.0%)	2 (2.6%)	0.245
Thrombolysis	12 (7.8%)	8 (5.3%)	4 (2.6%)	0.357
Adverse outcome§§	9 (5.9%)	3 (3.9%)	6 (7.9%)	0.327
PE-related death	3 (2.0%)	1 (1.3%)	2 (2.6%)	0.620
All-cause death	3 (2.0%)	1 (1.3%)	2 (2.6%)	0.620
In-hospital stay (days)	9 (6–13)	8 (6–12)	8 (5–13)	0.727
**Follow-up and long-term outcome**				
Longterm follow-up (days)	2968 (1186–2691)	2344 (1554–2986)	1927 (1097–2375)	0.013
Longterm mortality	28 (18.9%)	13 (17.6%)	15 (20.3%)	0.834
Recurrence of VTE	0 (0.0%)	0 (0.0%)	0 (0.0%)	1.000
CTEPH	6 (3.9%)	5 (6.5%)	1 (1.3%)	0.210

Statistical methods: Data are presented as absolute numbers (percentages) or medians (25–75th percentile), (n) refers to the number of patients with available data, p-values were calculated by the Mann–Whitney *U*-test (A) or Fisher’s Exact test (B).

*BMI* body mass index, *DVT* deep vein thrombosis, *PE* pulmonary embolism, *ESC* Euopean Sociey of Cardiology, *Spesi* simplified Pulmonary Embolism Severity Index, *CT* computed tomography, *TTE* transthoracic echocardiography, *RVD* right ventricular dysfunction, *hsTnT* high sensitive troponin T, *NT-proBNP* N-terminal propeptide of B-type natriuretic peptide, *GFR* glomerular filtration rate, *CRP* C-reactive protein, *VTE* venous thromboembolism, *CTEPH* chronic thromboembolic pulmonary hypertension.

*Within the last 4 weeks.

**Post partum within the last 6 weeks.

^†^Hypoxia was defined as methemoglobin oxygen saturation < 90% (regardless whether oxygen was given or not).

^††^RV dysfunction was defined as dilatation of the right ventricle (end-diastolic diameter > 30 mm from the parasternal view, or a right ventricle/left ventricle diameter ratio > 1.0 from the subcostal or apical views), combined with right atrial hypertension (absence of the inspiratory collapse of the inferior vena cava).

^§^Low risk was defined as absence of RV dysfunction and absence of troponin elevation; intermediate risk was defined as RV dysfunction and/or troponin elevation.

high risk was defined as haemodynamic instability.

^§§^Defined as mechanical ventilation, catecholamine administration, CPR or all-cause death within 30 days.

Significant values are in bold.

## Discussion

Acute pulmonary embolism remains a substantial and leading contributor to total mortality, even if in the last years the management and treatment of this preventable thrombotic disorder was evolving^2,9,10^. Patients with acute PE are characterized by a heterogeneous short-term and long-term prognosis; thus, estimation of the individual risk of death related to acute PE or from its associated complications is of paramount interest for initiating individual risk-adapted diagnostic and therapeutic strategies.

In the present study, we investigated the potential role of miRNA signatures in patients with PE and CTEPH. In summary, our findings suggest that miR-let7a plasma levels were strongly associated with an increased risk of CTEPH diagnosis during follow-up.

To date only a handful of studies investigated miRNA signatures in patients with PE. Nonetheless, these studies have shown that miRNAs may be used as diagnostic tools in the context of acute PE. Xiao et al. identified miR-134 as a potential biomarker for acute PE^11^. Another study by Zhou and colleagues observed increased circulating miR-28-3p levels in PE patients^12^. In a further study, Liu et al. found miR-221 as a novel diagnostic marker for PE and observed that plasma levels could be correlated with BNP, troponin I and D-dimer^13^. In 2015, Starikova et al. evaluated the miRNA expression profile in the plasma of 20 patients with a first unprovoked VTE and 20 age- and sex-matched healthy controls. This study revealed that five miRNAs were upregulated, and four miRNAs were downregulated in VTE patients versus controls^14^. In addition, other studies strengthened the clinical significance of specific miRNAs to serve as diagnostic markers for PE as it was shown that miRNAs may distinguish PE from other differential diagnosis such as myocardial infarction or non ST-segment elevation myocardial infarction^15,16^. The number of studies exploring the use of miRNAs as biomarkers in PE patients substantially increased during the past few years. However, most of the reports were case–control studies appearing to be limited by the small number of PE patients included and most did not use heparinase pre-treatment making interpretation of those studies difficult. To our knowledge, our current study is the first report identifying dysregulated circulating miRNAs in a larger patient cohort (n = 177) and using an optimized heparinase-pretreatment. In our study, we analyzed miRNA patterns in plasma samples derived from patients with acute PE via array-PCR-based profiling identifying novel blood-based biomarkers for the diagnosis of PE. We found significantly increased levels of miR-29a and significantly decreased plasma levels of miR-let7a and miR-720 in patients with PE suggesting that these miRNAs might serve as novel diagnostic tools. Neither miR-29a nor miR-720 correlated with the acute phase in patients with acute PE. Only miR-let7a predicted haemodynamic compromise in patients with acute PE and has not yet described in this context. In our validation cohort, we demonstrated the same expression pattern for miR-29a and miR-let7a regarding the outcome of death or recurrent event of acute VTE.

However, the expression of several haemostatic factors has been reported to be regulated by miRNAs including key procoagulant factors and inhibitors of the coagulation pathway^17^. Defective angiogenesis, incomplete thrombus revascularisation and fibrosis are considered critical pathomechanisms of CTEPH after PE^18^. The miR-29 family is known as key regulators of fibrosis in the heart and other organs. The members of the miR-29 family target at least 16 genes related to ECM (extracellular matrix), such as collagens, fibrillins, and elastin, and are known as key regulator of fibrosis by regulation of the profibrotic activities of fibroblasts and SMCs (smooth muscle cells). Previously, it was shown that transforming growth factor-beta (TGF-β)-mediated downregulation of miR-29 enhances profibrotic activities of mesenchymal-derived cells^19^. Angiogenesis and an intact angiogenic response after acute thrombosis or thromboembolism, are key mediators of normal thrombus resolution^20,21^. Direct upregulation of endothelial miR-29a by TGF-ß/Smad 4 signaling is a mediator of angiogenesis via increasing tube formation and human endothelial cell (EC) migration. In fresh thrombi TGF-ß-mRNA is increased compared to CTEPH specimens, suggesting TGF-ß mediated upregulated miR-29a expression in ECs to be involved in the early physiologic angiogenic response necessary for thrombus resolution. Previous reports have also found that miR-let‐7a plays a role in regulating angiogenesis^22^. A recent study demonstrated that miR-let‐7a regulated angiogenesis by concomitantly downregulating the TGF-ß-receptor type III (TGFBR3). Overexpression of miR-let‐7a or a knockdown of TGFBR3 in cell culture inhibited the tube formation and reduced migration rate^23^. In the present study, 3.4% patients with acute PE were diagnosed with CTEPH at follow-up. miR-29a and miR-720 levels were not considerable elevated in those patients and thus calculated patient-cohort optimized cut-off values were not associated with an increased risk for a diagnosis of CTEPH during follow-up. In contrast, we found that miR-let7a levels were significantly upregulated in patients at the time of PE diagnosis who diagnosed with CTEPH during follow-up compared to PE patients who did not develop CTEPH. Moreover, miR-let7a plasma concentrations above the calculated optimal cut-off value were associated with a 12.8-fold increased risk for the diagnosis of CTEPH at follow-up. In context with previous studies, which demonstrated that miR-let7a overexpression may contribute to defective angiogenesis and persistent vascular occlusion, our study results add relevant associations for miR-let7a for patients with acute PE with an increased risk for CTEPH. The present study has limitations that need consideration: first, although we were able to demonstrate an increased risk for subsequent diagnosis of CTEPH patients with pulmonary embolism and high miR-let7a plasma levels, further research is needed to determine whether miR-let7a may help in clinical decision making and prognostic assessment of individual patients. Second, CTEPH is a rare disease, therefore the number of CTEPH cases is limited and thus also the statistically power.

In summary, we found in our derivation and validation cohort significantly increased levels of miR-29a and significantly decreased plasma levels of miR-let7a in patients with an acute event of PE or recurrent VTE suggesting that these miRNAs might serve as novel diagnostic tools. Circulating miR-let7a levels were associated with haemodynamic compromise in patients with acute PE and additionally miR-let7a levels on admission were associated with high risk for the diagnosis of CTEPH during follow-up. Thus, miR-let7a might serve as a novel biomarker to identify patients with haemodynamic impairment and as a novel prediction marker for patients, who had a higher risk to develop CTEPH or for pre-existing CTEPH. Further studies are indispensable to evaluate if these miRNAs are specifically regulated in the acute phase of PE or also in the progression of the chronic disease.

## Methods

### Patients with acute pulmonary embolism

Consecutive patients aged ≥ 18 years with confirmed acute PE were prospectively included in a single center prospective cohort study (Pulmonary Embolism Registry of Göttingen, PERGO, supplemental Figs. 2 ,  3)) at the University Medical Center Goettingen, Germany. The study protocol has been described in detail elsewhere^24^. The study protocol was conducted in accordance with the amended Declaration of Helsinki and was approved by the local independent Ethic Committee of the Medical University Goettingen, Germany (AZ 14/6/10). All patients gave informed written consent for participation in the study.

Patients were excluded from the present study if PE was asymptomatic and/or an accidental finding obtained during the diagnostic workup for another suspected disease; or if consent for participation in the study was denied or withdrawn.

Thirty-day clinical follow-up data were obtained from all patients included in the study and an adverse 30-day outcome was defined as PE-related death, need for mechanical ventilation, cardiopulmonary resuscitation or the administration of catecholamines. Long-term follow-up data were collected 6 months after the initial PE event during a routine clinical follow-up (outpatient) visit as part of standard clinical care at the Clinic for Cardiology and Pulmonology, University Medical Center Goettingen, Germany or by contacting the treating general practitioner. Diagnosis of CTEPH was confirmed according to current guideline recommendations^25^. All study outcomes were independently adjudicated by two researchers.

### Control group

The control group consisted of sex- and age-matched patients (based on SPSS case–control matching) apparently healthy volunteers without a history of chronic disease.

### Plasma sampling and RNA isolation

Venous blood samples for subsequent biomarker measurements were collected from patients with acute PE. Blood samples were processed using standard operating procedures, immediately stored at − 80 °C and analysed in batches after a single thaw. Routine laboratory parameters were measured as part of the clinical routine at the Department of Clinical Chemistry of the University Medical Centre Goettingen, Germany. For RNA isolation the miRNeasy Serum/Plasma Kit (Qiagen) was used according to the manufacturer´s instructions. For subsequent normalization synthetic Caenorhabditis elegans miR-39 (5 μl of 1 fmol/μl) was added as spike-in control during RNA isolation to the Qiazol/chloroform/plasma mixture^26^.

### Heparinase pre-treatment

To remove heparin in RNA samples after RNA isolation, samples were treated with heparinase according to previous studies^27^. Briefly, 3.5 µl of RNA was mixed with 0.19 µl RNase inhibitor (20 U/µl), 1 µl 10 × RT buffer (Applied Biosystem), 0.9 µl MgCl_2_ (25 mM, Qiagen) and 0.3 µl heparinase (1U/µl) and filled up to 10 µl with RNase-free H_2_O. The reaction mixture was incubated for 1 h at 25 °C.

### TaqMan ArrayCard miRNA screening

A panel of 754 miRNAs was analysed in plasma of 20 out of 177 randomly selected patients with acute PE and 20 sex- and age-matched healthy volunteers via human TaqMan array MicroRNA A + B Cards Set (Applied Biosystems) following the manufacturer’s recommendation. Prior to polymerase chain reactions (PCR), two steps consisting of reverse transcription (RT) and pre-amplification were conducted. The Megaplex PreAmp Primers, consisting of two pools of gene-specific forward and reverse primers (Pool A and Pool B) were used to enable the unbiased pre-amplification of the miRNA cDNA target by PCR.

### Validation of findings using quantitative real-time PCR

After heparinase treatment, TaqMan MicroRNA Reverse Transcription Kit (Applied Biosystems) was used for cDNA synthesis following the manufacturer´s instructions. Primers hsa-miR-720, hsa-miR-29a, hsa-let7a and cel-miR-39 were used in this study. Quantification of specific miRNA levels was performed via quantitative RT-PCR using TaqMan MicroRNA Assays (Applied Biosystems). The qPCR was performed in a 384-well PCR plate using the ViiA™ 7 Real-Time PCR System (Thermo Fisher Scientific). The qRT-PCR reaction mix was run at 95 °C for 15 min, followed by 45 cycles of denaturation at 95 °C for 15 s and combined annealing/elongation at 60 °C for 1 min. Relative quantification was performed using the Delta-delta CT method^28^.

### External validation in an independent cohort

MicroRNAs of interest were evaluated in relation to the combined endpoint of recurrent event or death in an independent external cohort, the Genotyping and Molecular Phenotyping in Venous ThromboEmbolism (GMP-VTE) project^29^. GMP-VTE is an investigator-initiated, prospective, multi-centre cohort study of VTE patients enrolled in the acute phase, with collection of biomaterial and long-term follow-up (up to 8 years). Quantitative determination of circulating miRNAs was performed in a subsample of 181 individuals. Small RNA isolation and library preparation was performed by GenXPro GmbH (Frankfurt am Main, Germany) in 200 µl of once-thawed ethylenediaminetetraacetic acid (EDTA)-treated plasma using the NucleoSpin RNA Mini kit (Machery & Nagel, Düren, Germany), and Small RNA Next Generation Sequencing (NGS)-libraries were prepared using the TrueQuant smallRNA-Seq kit (GenXPro GmbH) according to the manual of the respective manufacturers. Sequencing was performed on an Illumina NextSeq500 instrument with 75 cycles for sequencing. Low quality reads were trimmed and adapters were removed by the ‘cutadapt’ algorithm. PCR duplicates were removed based on the TrueQuant molecular identifiers, i.e. poly-N nucleotides incorporated in the template molecules prior to amplification such that each has a unique sequence (GenXPro GmbH). Reads were mapped and annotated using the OmiRas pipeline. Read data were normalized to counts per million (CPM) format. Data were subsequently transformed to normality by inverse normal transformation^30^.

### Statistics

The Fisher´s exact test or χ^2^ test was used to compare categorical variables, which were expressed as absolute numbers or percentage. Continuous variables were found not to follow a normal distribution when tested with the modified Kolmogorov–Smirnov test (Lilliefors test); therefore, these variables are expressed as medians with the corresponding interquartile range (IQR) and were compared using the unpaired Mann–Whitney-U test. Receiver operating characteristics (ROC) curve analysis was performed to determine the area under the curve (AUC)^31^. Youden-index quantification was used to identify the optimal cut-off values for miRNA-based prediction of the study outcomes. Comparison of the prognostic performance of biomarker levels was performed by calculation of sensitivity, specificity, positive predictive value (PPV) and negative predictive value (NPV), and positive and negative likelihood ratios (LR). The prognostic relevance of biomarker cut-off values as well as single predictors with regard to study outcomes was tested using univariable logistic regression analysis and presented as Odds ratios (OR) with the corresponding 95% confidence intervals (CI). A two-sided significance level of α < 0.05 was defined appropriate to indicate statistical significance. All statistical analyses were performed using the SPSS software (version 21.0, SPSS Inc., Chicago, Illinois, USA).

For analyzing the regulation of miRNAs between the different patient groups an unpaired student´s t-test was used. Analysis and illustrative presentations of expression data was conducted by Graphpad prism.

### Supplementary Information

Supplementary Information.

## Data Availability

The datasets generated during and/or analysed during the current study are available in the GEO repository with the accession number GSE247365 and the link https://www.ncbi.nlm.nih.gov/geo/query/acc.cgi?acc=GSE247365.
