# Supplementary material for: Circulating miR-let7a levels predict future diagnosis of chronic thromboembolic pulmonary hypertension
Source: Sci Rep. 2024 Feb 24;14:4514. doi: 10.1038/s41598-024-55223-1 (PMC10894210; doi:10.1038/s41598-024-55223-1)
Supplement: Supplementary file 1 — Supplementary Information. [file 41598_2024_55223_MOESM1_ESM.pdf]

Circulating miR-let7a levels predict future diagnosis of chronic thromboembolic pulmonary hypertension

Author list:

Franziska Kenneweg[1,2#], Lukas Hobohm[3,4#], Claudia Bang[1], Shashi K. Gupta[1], Ke Xiao[1], Sabrina Thum[1], Vincent ten Cate[5,6], Steffen Rapp[5,7], Gerd Hasenfuß[8], Philipp Wild[5,6,7,8], Stavros Konstantinides[4], Rolf Wachter[9,10], Mareike Lankeit[4,11], Thomas Thum[1,2*]

Affiliations:

[1]Institute of Molecular and Translational Therapeutic Strategies (IMTTS), Hannover Medical School, Hannover, Germany
[2]REBIRTH Excellence Cluster, Hannover Medical School, Hannover, Germany
[3]Department of Cardiology, University Medical Center Mainz, Germany
[4]Center for Thrombosis and Hemostasis (CTH), University Medical Center Mainz, Germany
[5]Preventive Cardiology and Preventive Medicine, Department of Cardiology, University Medical Center Mainz, Germany
[6]Clinical Epidemiology and Systems Medicine, Center for Thrombosis and Hemostasis (CTH), Mainz, Germany
[7]German Cardiovascular Research Centre (DZHK), partner site Rhine Main, Mainz Germany
[8]Institute of Molecular Biology (IMB), Mainz, Germany.
[9]Clinic of Cardiology and Pneumology, Heart Center, University Medical Center Goettingen, Germany
[10]Clinic and Policlinic for Cardiology, University Hospital Leipzig, Germany
[11]Department of Internal Medicine and Cardiology, Campus Virchow Klinikum (CVK), Charité – University Medicine Berlin, Germany

# F.K. and L.H. contributed equally and share first authorship

* corresponding author

Supplemental Data

Supplemental Figure 1: Matched Data of miR-let7a, miR-29a and miR-720 in patients with acute pulmonary embolism. The three promising miRNAs were validated in a patient cohort of 177 PE and 177 healthy patients by qPCR. (A) miR-let7a was found to be significantly decreased, (B) miR-29a to be significantly increased and (C) miR-720 to be significantly decreased in patients with pulmonary embolism (PE).; ** p≤0.01; paired Student´s t-test of patients with PE and specific sex- and age-matched healthy controls

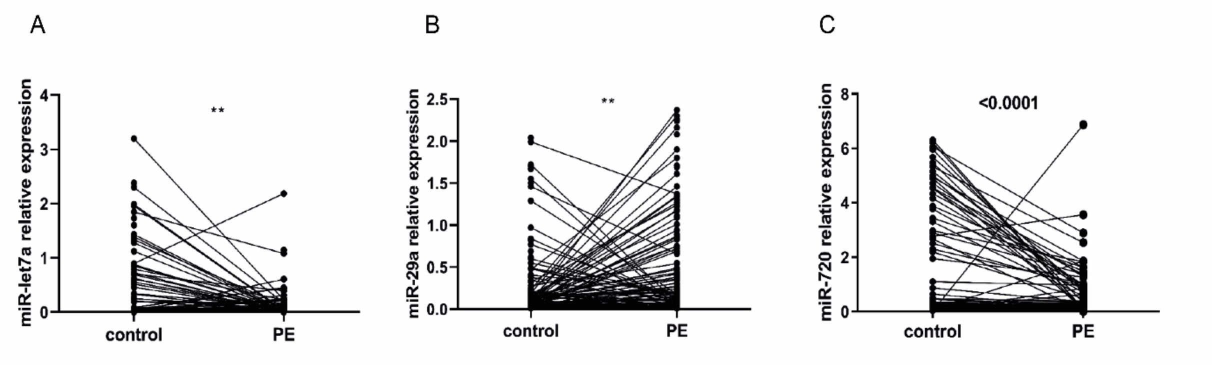

Supplemental Figure 2: Patient cohort for miRNA screening. N=20 patients with pulmonary embolism (n=10 low-risk and n=10 intermediate risk patients) were subjected to miRNA screening.

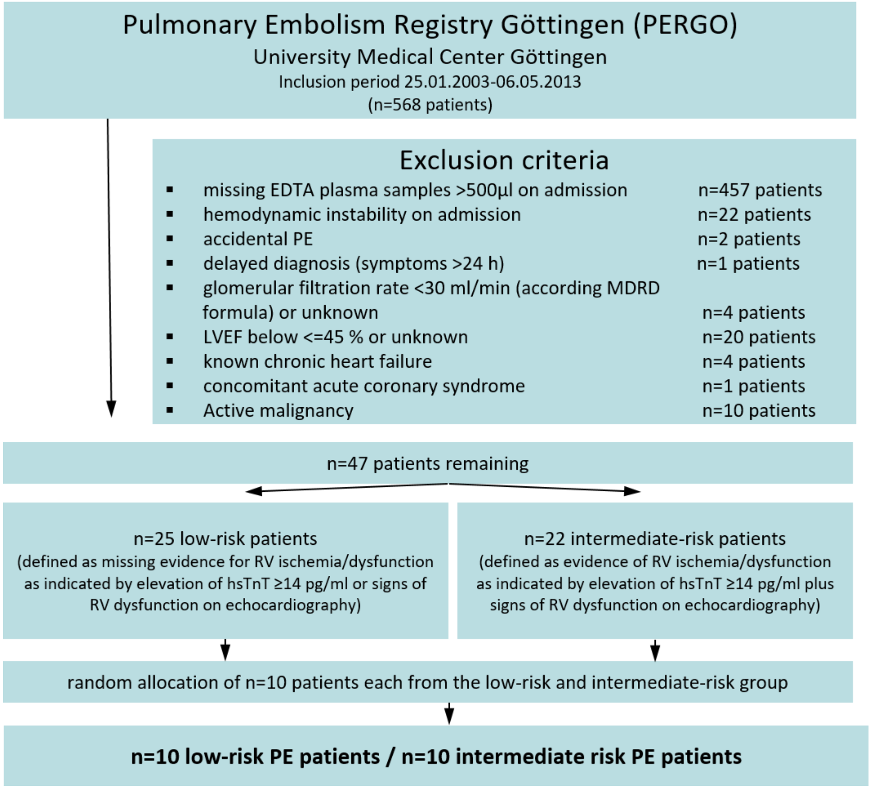

Supplemental Figure 3: Patient cohort for validation experiments. Plasma of n=177 patients with pulmonary embolism were used for validation studies.

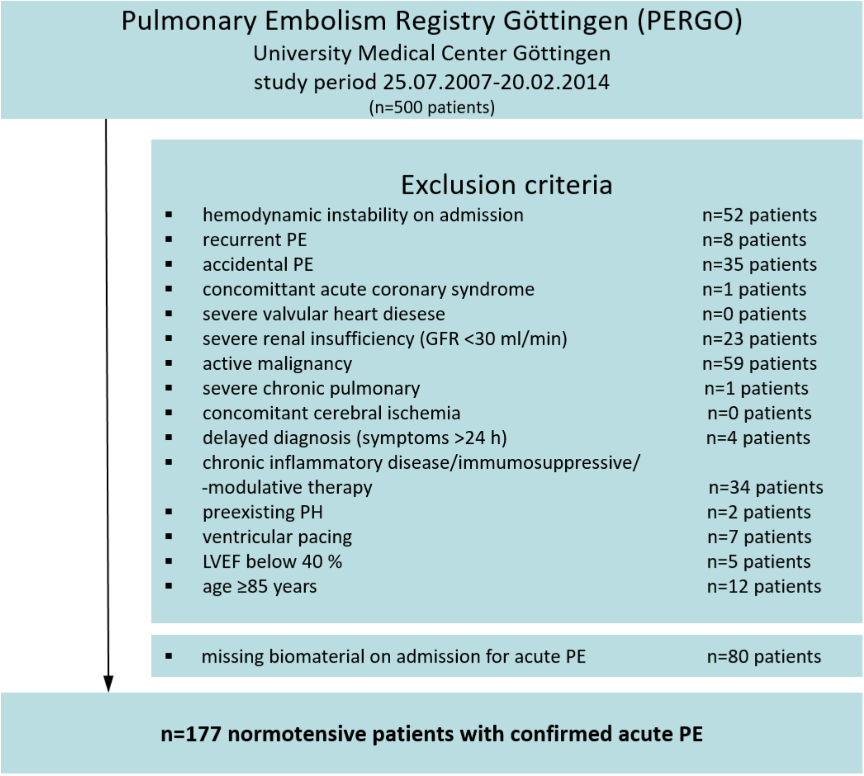

Supplemental Table 1: Top 10 of deregulated miRNAs

Up	Down
miR-564	miR-122
miR-1290	miR-483
miR-939	miR-23b
miR-874	miR-206
miR-629	miR-29b
miR-193a	miR-1301
miR-660	miR-98
miR-125a	miR-17
miR-720	miR-26a
miR-29a	let-7a
